# Assessing Guideline Knowledge Alignment of Large Language Models in Ophthalmology: A Preclinical Benchmarking Study on KLEx Evidence‐Based Guidelines

**DOI:** 10.1155/joph/4163980

**Published:** 2026-08-29

**Authors:** Hongxia Lu, Yan Huo, Ruisi Xie, Zhengyuan Qu, Yutong Li, Shengjin Wang, Haohan Zou, Yan Wang

**Affiliations:** ^1^ Clinical College of Ophthalmology, Tianjin Medical University, Tianjin, China, tijmu.edu.cn; ^2^ Tianjin Eye Hospital, Tianjin Key Lab of Ophthalmology and Visual Science, Tianjin Eye Institute, Nankai University Affiliated Eye Hospital, Tianjin, China, oio.cn; ^3^ School of Medicine, Nankai University, Tianjin, China, nankai.edu.cn; ^4^ Department of Electronic Engineering, Tsinghua University, Beijing, China, tsinghua.edu.cn; ^5^ HuaHui Jian AI Tech Ltd, Tianjin, China

**Keywords:** DeepSeek-V4, evidence-based guidelines, GPT-5.5 Instant, keratorefractive lenticule extraction, large language models

## Abstract

**Purpose:**

To evaluate the guideline knowledge alignment of two large language models (LLMs), GPT‐5.5 Instant and DeepSeek‐V4, and to determine their preclinical reliability as reference tools in refractive surgery.

**Methods:**

Using the 38 evidence‐based recommendations of the international keratorefractive lenticule extraction (KLEx) guidelines as the gold standard, both LLMs were evaluated in their default configurations. Two ophthalmologists independently assessed the clinical safety and medical accuracy of the model responses using a 5‐point Likert scale (1–5 points). Agreement between each model’s recommendation strength and the guideline was quantified by intraclass correlation coefficient (ICC), structural reliability by the DISCERN scale, and readability by the Flesch Reading Ease (FRE) and Flesch–Kincaid Grade Level (FKGL) indices.

**Results:**

Likert ratings did not differ between GPT‐5.5 Instant (4.96 ± 0.206) and DeepSeek‐V4 (4.89 ± 0.385; *p* = 0.134). Across 114 independent generations, the ICC for agreement with the guideline was 0.884 (95% CI, 0.836–0.918) for GPT‐5.5 Instant and 0.739 (0.640–0.813) for DeepSeek‐V4 (both *p* < 0.001). DISCERN scores were 70.18 ± 5.16 and 68.05 ± 5.41 (*p* = 0.085); FRE, 9.93 ± 8.33 and 3.74 ± 5.24 (*p* < 0.001); and FKGL, 16.07 ± 2.21 and 18.95 ± 1.96 (*p* < 0.001).

**Conclusion:**

Both models aligned closely with the guidelines, with significant concordance in GRADE‐based recommendation strength. They may serve as preclinical reference tools in refractive surgery but not as a substitute for specialist judgment.

## 1. Introduction

By 2050, the global myopia population is expected to increase to 4.758 billion, accounting for half of the total population [1], leading to a rising demand for myopia refractive surgery. Keratorefractive lenticule extraction (KLEx), as one of the current mainstream refractive surgeries, has rapidly gained popularity in recent years, with over 8 million procedures performed worldwide by the end of 2023 [2]. Compared to photorefractive keratectomy (PRK) and laser in situ keratomileusis (LASIK), KLEx has certain advantages in preserving the natural structure and biomechanical integrity of the cornea and reduces the risk of postoperative complications, such as dry eye, caused by the production of corneal flaps or larger incisions [3]. However, KLEx has a longer learning curve and requires ophthalmologists to receive long‐term education and clinical training [4]. Especially, with the development of precision medicine, issues such as preoperative risk assessment and surgical plan design pose new challenges for clinicians [5]. As a new technology, clinical doctors are eager for technical guidance to reduce complications and improve surgical safety, ultimately optimizing patient outcomes [6, 7].

Large language models (LLMs) can integrate dispersed clinical knowledge to support evidence‐based decision‐making [8–10], with demonstrated utility in previsit triage, electronic health record analysis, and interpretation of diagnostic reports [11–13]. Whether this extends to narrow surgical subspecialties, however, remains largely untested. We, therefore, evaluated GPT‐5.5 Instant (OpenAI) and DeepSeek‐V4 (DeepSeek), representative of current proprietary and open‐weight models in clinical use [14, 15].

Recently, KLEx International Guidelines have been developed based on evidence‐based medicine [16], which have high authority in the professional field, providing a key opportunity to address this issue. They can effectively help validate the ability assessment of LLM in the clinical practice of refractive surgery. Therefore, this study aims to systematically evaluate the performance of GPT‐5.5 Instant and DeepSeek‐V4 in analyzing and answering 38 KLEx surgery–related questions based on international guidelines, focusing on the accuracy, reliability, depth of reasoning, readability, and consistency with existing clinical standards. We frame guideline alignment as a preclinical prerequisite—baseline evidence that should be established before LLMs are evaluated in live clinical workflows.

## 2. Methods

This study uses the 38 recommendations of the KLEx evidence‐based guidelines as the gold standard, covering the most common and critical clinical issues in KLEx surgery. The guidelines were developed by 44 global experts and associated teams to provide standardized protocols for KLEx. They adhere to the methodology of the World Health Organization (WHO), the Grading of Recommendations, Assessment, Development, and Evaluations (GRADE) system, and a Delphi expert‐consensus approach. Evidence was synthesized from more than 250,000 publications, yielding 38 evidence‐based recommendations spanning three domains: indications, surgical planning and techniques, and postoperative monitoring and management of complications [2]. The full recommendation set is provided in Supporting Information 1.

To improve the clarity and clinical relevance of the content presented to each model, each of the 38 evidence‐based recommendations was reformulated as a standardized clinical question. For organization, these 38 questions were grouped into the 15 thematic clinical domains defined by the KLEx guidelines, with the 38 recommendations retained as the unit of analysis throughout. During testing, each standardized question was submitted individually to each model in a new session using standardized prompts (see Supporting Information 2 for details). Because LLMs may return different responses to the same input, each question was submitted in three independent sessions, yielding 114 responses per model (228 in total). Both models were accessed through their standard web interfaces under default settings.

**FIGURE 1:**
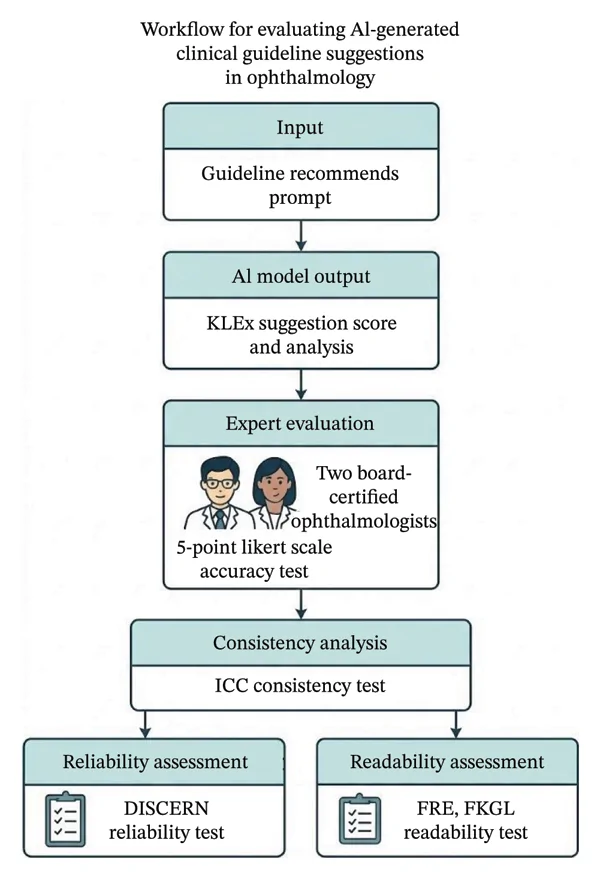
Study design flowchart. Abbreviations: KLEx, keratorefractive lenticule extraction; ICC, intraclass correlation coefficient; FRE, Flesch Reading Ease; FKGL, Flesch–Kincaid Grade Level.

The primary objective was to assess whether the models’ responses aligned with the evidence‐based guidelines rather than to evaluate literature‐retrieval ability; the models were, therefore, not required to supply references. The overall workflow is shown in Figure 1.

The responses generated by the models were independently evaluated by two attending ophthalmologists (Hongxia Lu and Yan Huo) and rated using a standardized 5‐point Likert scale (scoring criteria are provided in the electronic supporting information). To eliminate evaluator bias, all LLM‐generated responses were extracted by a third independent researcher (Ruisi Xie), stripped of model‐specific identifiers (e.g., Markdown formatting and logos) and presented in standardized plain text. Evaluators remained completely blinded to the source of the responses. To strictly evaluate the clinical safety and medical accuracy of the LLMs rather than mere linguistic fluency, the 5‐point Likert scale was specifically anchored to clinical risks and the KLEx evidence‐based guidelines. The two evaluating ophthalmologists were instructed to adhere to the following rigorous grading rubric:

1. Strongly Disagree: The response contains severe medical errors, fundamentally contradicts the KLEx guidelines, and poses a significant risk of patient harm, surgical complications, or severe mismanagement if adopted in clinical practice.
2. Disagree: The response contains major inaccuracies or misinterprets the guidelines. While not immediately life‐threatening, it provides unsafe or flawed advice that could lead to suboptimal surgical planning or adverse patient outcomes.
3. Neither Agree nor Disagree: The response is noncommittal, overly generalized, or lacks specific actionable information related to KLEx. It neither actively harms nor practically assists clinical decision‐making.
4. Agree: The response is fundamentally accurate and safe and aligns well with the KLEx guidelines, serving as a reliable preclinical reference, though it may lack exhaustive detail or minor clinical nuances.
5. Strongly Agree: The response perfectly aligns with the gold‐standard KLEx guidelines. It is exceptionally accurate, comprehensively safe, and provides directly actionable and rigorous insights for a refractive surgeon.

In the evaluation process, if there is disagreement between two reviewers, the research team will invite a senior physician with over 30 years of experience (YW) to conduct an assessment. Through discussion and based on the established rating criteria, a final consensus will be reached to ensure the objectivity and consistency of the ratings. This study employs the intraclass correlation coefficient (ICC) to assess the consistency between the ratings of the two models and those of the expert panel. The ICC value ranges from 0 to 1, with higher values indicating better consistency.

To assess structural reliability, the DISCERN instrument, originally developed for patient‐facing health information, was applied by the same panel of physicians who audited the Likert assessments. The FRE index and the FKGL were used to assess linguistic complexity and readability. The DISCERN instrument assesses the objectivity and completeness of information on medical treatments. It comprises three sections: the first (8 items) evaluates the reliability of information sources; the second (7 items) addresses the specifics of the treatment options; and the third provides an overall quality rating. Total DISCERN scores range from 16 to 80 and are categorized as Excellent (63–80), Good (51–62), Fair (39–50), Poor (27–38), or Very Poor (16–26) [17]. Because the objective was to assess concordance with the guideline rather than literature‐retrieval ability, and the models were not asked to supply references, the source‐related items were scored on the accuracy and authority of the content relative to the source guideline rather than on the presence of formatted citations. FRE scores range from 0 to 100, with higher values indicating greater readability; raw FRE values below 0 were truncated to 0, consistent with the nominal 0–100 range of the index; FKGL estimates the U.S. school grade level required to comprehend the text, with higher values indicating greater complexity.

This study was conducted in a controlled computer environment (Microsoft Windows 11 operating system; Microsoft Edge version 134.0.3124.51). On June 13, 2026, two LLMs were evaluated: DeepSeek‐V4 (DeepSeek‐AI, Hangzhou, China) and GPT‐5.5 Instant (OpenAI, San Francisco, USA).

### 2.1. Statistical Analysis

To account for model stochasticity, each of the 38 recommendations was queried in triplicate, yielding 114 generations per model, and the analysis was performed at two levels. At the individual‐generation level (*n* = 114 per model), the agreement between each model’s GRADE‐based recommendation strength and the guideline‐defined recommendation strength was assessed using the single‐measures, absolute‐agreement ICC [ICC (2, 1)]. At the recommendation level, the triplicate outputs were averaged for between‐model comparisons (*n* = 38 paired observations). The Kolmogorov–Smirnov test was used to assess the normality of the paired differences. Normally distributed variables were compared using the paired *t*‐test, and variables that did not follow a normal distribution were analyzed using the Wilcoxon signed‐rank test. Quantitative variables are presented as mean ± standard deviation or median (Q1, Q3). Inter‐rater reliability of the DISCERN evaluations between the two attending ophthalmologists was assessed using the ICC. All statistical analyses were performed using IBM SPSS Version 27.0 (IBM Corp., Armonk, NY, USA), with a significance level set at *p* < 0.05.

## 3. Results

### 3.1. Accuracy of LLMs

There was a high level of inter‐rater agreement between the two attending ophthalmologists during the evaluation process. For the 5‐point Likert scale, the ICC for GPT‐5.5 Instant was 0.826 (95% CI, 0.757–0.876) and for DeepSeek‐V4, it was 0.896 (95% CI, 0.852–0.927), indicating robust consistency. In the Likert evaluation (Table 1), GPT‐5.5 Instant received a rating of 5 on 109 responses (95.61%) and a rating of 4 on 5 responses (4.39%). DeepSeek‐V4 received a rating of 5 on 105 responses (92.11%), a rating of 4 on 6 responses (5.26%), and a rating of 3 on 3 responses (2.63%). Neither model received a rating of 1 or 2.

**TABLE 1:** The scores of the answers to the 114 questions provided by the LLMs are presented on a Likert scale.

Likert scale	GPT‐5.5 Instant (*n*)	DeepSeek‐V4 (*n*)
5	109	105
4	5	6
3	—	3
2	—	—
1	—	—

As shown in Table 2, there was no statistically significant difference in the Likert scores between GPT‐5.5 Instant (4.96 ± 0.206) and DeepSeek‐V4 (4.89 ± 0.385, *p* = 0.134).

**TABLE 2:** Mean ± standard deviation (SD) scores of the two LLMs in total question categories.

LLMs	Total	*p*
GPT‐5.5 Instant (Mean ± SD)	4.96 ± 0.206	0.134
DeepSeek‐V4 (Mean ± SD)	4.89 ± 0.385	

*Note:* Wilcoxon signed‐rank test.

### 3.2. Consistency of LLMs

Table 3 presents the agreement between each model’s expressed recommendation strength and the guideline‐defined recommendation strength across the 114 generations. Agreement for GPT‐5.5 Instant was (ICC = 0.884; 95% CI, 0.836–0.918; *p* < 0.001) and for DeepSeek‐V4 (ICC = 0.739; 95% CI, 0.640–0.813; *p* < 0.001). Both models showed statistically significant alignment with the GRADE‐based recommendation strength established by the guidelines.

**TABLE 3:** Comparison of consistency scores of the LLMs.

LLMs	ICC score	95% CI	*p* value
GPT‐5.5 Instant vs. Consensus	0.884	0.836–0.918	< 0.001
DeepSeek‐V4 vs. Consensus	0.739	0.640–0.813	< 0.001

*Note:* Evidence‐based Guidelines’ recommend score.

### 3.3. Reliability and Readability of LLMs

To illustrate clinical utility, Table 4 presents two representative responses in refractive‐surgery scenarios. Inter‐rater agreement for the DISCERN evaluations was high (GPT‐5.5 Instant: ICC = 0.927, 95% CI, 0.896–0.950; DeepSeek‐V4: ICC = 0.955, 95% CI, 0.935–0.969), indicating stable and reliable scoring. GPT‐5.5 Instant scored numerically higher than DeepSeek‐V4, but the difference was not statistically significant (70.18 ± 5.16 vs. 68.05 ± 5.41; *p* = 0.085); both fell within the “Excellent” band.

**TABLE 4:** Representative examples of LLM responses in refractive surgery scenarios.

Thematic domain (Rec. no.)^a^	Clinical question (abbreviated)	GPT‐5.5 Instant—summary of key response content	DeepSeek‐V4—summary of key response content	Clinical assessment^b^
Surgical planning (Rec. 5)	Is PTA in KLEx mainly based on the removed lenticule thickness?	Rated the statement directionally correct but imprecise; scored +1.	Rated the statement incorrect, noting PTA = (cap + lenticule)/CCT; scored −1.	Guideline +1. GPT‐5.5 Instant matched; DeepSeek‐V4 over‐corrected a technical detail into a negative grade, though its explicit formula reflected greater transparency.
Astigmatism correction (Rec.12)	Is the correctable astigmatism range −0.25–−5.00 D, with weaker correction at higher targets?	Rated accurate and guideline‐aligned; scored +1.	Rated core claim correct but flagged −0.25 D bound as lacking consensus; scored 0.	Guideline +1. GPT‐5.5 Instant matched; DeepSeek‐V4 down‐graded over an unestablished detail, again over‐weighting caveats relative to the guideline.
Delayed visual recovery (Rec. 35)	Is the multistep management of delayed visual recovery appropriate?	Rated sound but down‐weighted visual‐function training as weakly evidenced; scored +1.	Rated fully guideline‐concordant; scored +2 (matched).	Guideline +2. DeepSeek‐V4 matched; GPT‐5.5 Instant under‐graded by over‐weighting one component’s limited evidence.

Abbreviations: CCT, central corneal thickness; KLEx, keratorefractive lenticule extraction; LLM, large language model; PTA, percentage of tissue alteration.

^a^Recommendation numbers correspond to Supporting Information 1.

^b^Scores follow the guideline’s GRADE‐based scale (−2–+2); responses shown are author summaries of the model outputs.

Table 5 summarizes the readability and text complexity of the generated responses. The two models differed significantly on both indices: FRE scores were 9.93 ± 8.33 for GPT‐5.5 Instant and 3.74 ± 5.24 for DeepSeek‐V4 (*p* < 0.001), and the corresponding FKGL scores were 16.07 ± 2.21 and 18.95 ± 1.96, respectively (*p* < 0.001). Responses from both models fell within the “very difficult” range (FRE < 30) and corresponded to a graduate reading level (FKGL ≈ 16–19, equivalent to approximately 16–19 years of education), consistent with the specialized, clinician‐facing nature of the source guidelines.

**TABLE 5:** Comparison of reliability and readability mean scores of the LLMs.

LLMs	Reliability	Readability	
	DISCERN score, mean ± SD	Flesch reading ease score, mean ± SD	Flesch–Kincaid grade level, mean ± SD
GPT‐5.5 Instant	70.18 ± 5.16	9.93 ± 8.33	16.07 ± 2.21
DeepSeek‐V4	68.05 ± 5.41	3.74 ± 5.24	18.95 ± 1.96
*p* value	0.085	< 0.001	< 0.001

*Note:* Calculated using the paired *t*‐test (data presented as mean ± SD).

## 4. Discussion

This study evaluated the accuracy, consistency, reliability, and readability of GPT‐5.5 Instant and DeepSeek‐V4 in interpreting evidence‐based guidelines for corneal refractive surgery. Both models generated responses highly concordant with the KLEx recommendations, showing comparably high Likert ratings (4.96 ± 0.21 vs. 4.89 ± 0.39; *p* = 0.134) and DISCERN scores within the “Excellent” range (70.18 ± 5.16 vs. 68.05 ± 5.41; *p* = 0.085). Agreement with the guideline‐defined strength of recommendation was good for GPT‐5.5 Instant (ICC = 0.884) and moderate for DeepSeek‐V4 (ICC = 0.739). A significant between‐model difference was observed only in readability (FRE: 9.93 ± 8.33 vs. 3.74 ± 5.24; FKGL: 16.07 ± 2.21 vs. 18.95 ± 1.96; both *p* < 0.001), with both models producing text at a graduate reading level. These findings support the use of either model as a preclinical reference tool in corneal refractive surgery rather than as a substitute for expert judgment.

LLMs are increasingly used at the point of clinical consultation, changing how clinical information is retrieved and synthesized [18, 19]. Not only are patients increasingly turning to these systems for previsit guidance, but junior doctors and medical students are also adopting them as explanatory aids for clinical reasoning and self‐directed learning [20]. However, the capability of LLMs to handle specialized medical inquiries remains unclear, and there is a lack of systematic evaluation regarding the alignment between LLM‐generated responses and established clinical guidelines. This gap directly impacts the safety and efficacy of using LLMs as sources of professional medical advice for both clinicians and patients. Therefore, this study systematically evaluates the alignment of GPT‐5.5 Instant and DeepSeek‐V4 with evidence‐based ophthalmology guidelines to determine the clinical accuracy, consistency, and reliability of their responses, thereby providing an evidence‐based basis for the appropriate application of LLMs in real‐world clinical scenarios.

GPT‐5.5 Instant is OpenAI’s latency‐optimized default model, released in May 2026 with an emphasis on factual reliability, producing 52.5% fewer hallucinated claims than its predecessor on high‐stakes medical, legal, and financial prompts in internal evaluations [21]. DeepSeek‐V4, released in June 2026 under a permissive MIT license, is an open‐weight Mixture‐of‐Experts (MoE) model whose flagship variant carries 1.6 trillion total parameters, activates approximately 49 billion per token, and supports a one‐million‐token context window [15, 22]. Despite these differences in architecture and training, the two models interpreted the clinical guidelines comparably, with similar Likert ratings (*p* = 0.134).

Although both models achieved high accuracy, they differed in how faithfully they tracked the graded recommendations of the guideline. The two ICCs fell into different interpretive bands, good versus moderate, and their 95% confidence intervals did not overlap (0.836–0.918 vs. 0.640–0.813). The representative cases in Table 4 indicate that this difference was systematic rather than random. DeepSeek‐V4 lost concordance where it applied its own appraisal of the evidence in place of the guideline’s, for example by downgrading recommendations over the exact definition of percentage tissue altered and over an astigmatism limit it judged insufficiently established, whereas GPT‐5.5 Instant more consistently followed the codified GRADE synthesis. In effect, DeepSeek‐V4 tended to revise the guideline rather than reproduce it. Even where these revisions were individually reasonable, a reference tool is expected to reflect the guideline faithfully rather than rewrite it, which made DeepSeek‐V4 the less faithful of the two for this application. Notably, every discordance ran in the conservative direction, toward weaker endorsement, so the clinically dangerous failure mode of overclaiming was not observed. The DISCERN scores differed by only 2.13 points (*p* = 0.085), indicating similar reliability in conveying KLEx‐related information.

GPT‐5.5 Instant produced significantly more readable text than DeepSeek‐V4 on both indices (*p* < 0.001) although both outputs required graduate‐level reading proficiency. Because clinical practice guidelines are rich in specialized terminology and complex syntax, LLMs tend to replicate these patterns, preserving domain‐specific terminology and mirroring syntactic style [23]. While this maintains terminological fidelity, it limits readability for nonspecialist audiences. For the clinician‐facing use examined here, this register is appropriate, and the readability ceiling becomes a constraint only when outputs are repurposed for patient education [24]. Simple prompt‐level instructions specifying a target reading level have been shown to bring LLM‐generated patient materials substantially closer to recommended thresholds [25, 26], provided that simplified outputs are audited to ensure that readability gains do not compromise clinical accuracy.

As an open‐weight model under a permissive MIT license, DeepSeek‐V4 offers advantages in deployment cost, local operability, and data governance [15], which carry particular weight where institutional or regulatory constraints preclude routing clinical queries to external servers. GPT‐5.5 Instant, by contrast, offers lower latency and, in the present data, higher concordance with the guideline’s recommendation strength. The choice between them may therefore depend on whether users’ priorities favor data governance and customization or maximal fidelity to codified standards.

This study has several limitations. Because model performance is version dependent, these findings represent a snapshot of the specific model versions evaluated on the stated date and may not transfer to earlier or later versions. Second, this was a text‐based, single‐turn preclinical benchmark that did not assess multiturn interactions, real clinical workflows, or the usability of responses to patient inquiries. Third, to reflect routine accessibility, we evaluated the default configurations of both models rather than premium tiers, so performance under optimized settings may be higher. Future work will address these gaps through longitudinal, multiturn evaluation in real clinical scenarios.

## 5. Conclusion

Both GPT‐5.5 Instant and DeepSeek‐V4 showed high accuracy and significant agreement with expert‐defined recommendation strengths for KLEx surgery. Given their graduate‐level readability and the continued need for human validation, they are best used as assistive preclinical references in keratorefractive surgery rather than as autonomous decision‐makers.

**Nomenclature**
FKGL: Flesch–Kincaid grade level
FRE: Flesch reading ease indices
GRADE: Grading of recommendations, assessment, development, and evaluations
Guideline: Evidence‐based guidelines for keratorefractive lenticule extraction surgery
ICC: Intraclass correlation coefficient
KLEx: Keratorefractive lenticule extraction
LASIK: Laser in situ keratomileusis
LLMs: Large language models
PRK: Photorefractive keratectomy
WHO: World Health Organization

## Author Contributions

Hongxia Lu: conceptualization, investigation, formal analysis, writing–original draft, writing–review and editing, and visualization; Yan Huo: conceptualization, investigation, formal analysis, writing–review and editing, and visualization; Ruisi Xie: methodology, investigation, and project administration; Zhengyuan Qu: methodology, investigation, and project administration; Yutong Li: methodology, investigation, and project administration; Shengjin Wang: methodology, investigation, and project administration; Haohan Zou: writing–review and editing; Yan Wang: writing–review and editing and supervision. Hongxia Lu and Yan Huo contributed equally to this study. All authors provided critical revision of the manuscript. All authors attest that they meet the current ICMJE criteria for authorship.

## Funding

The research was supported by the National Program on Key Research Project of China (2022YFC2404502), National Natural Science Foundation of China (82271118), Tianjin Health Research Project (no. TJWJ2024QN076), and Tianjin Key Medical Discipline Construction (TJYXZDXK‐3‐004A‐3).

## Ethics Statement

The study protocol was submitted to our institutional review board for assessment. Because the study was conducted entirely in a computational environment on large language models and involved no human participants, animals, biological samples, or identifiable patient information, the board determined that it was exempt from ethical review. The participating ophthalmologists contributed solely as coinvestigators providing the technical reference standard, in accordance with ICMJE authorship criteria.

## Consent

The authors have nothing to report.

## Conflicts of Interest

The authors declare no conflicts of interest.

## Supporting Information

Additional supporting information can be found online in the Supporting Information section.

## Supporting information


**Supporting Information 1** Supporting Information 1. The full set of 38 recommendations from the KLEx evidence‐based guidelines.

**Supporting Information 2** Supporting Information 2. Standardized prompts for conversations on each clinical issue with LLM.

## Data Availability

The data that support the findings of this study are available from the corresponding authors upon reasonable request.
