# Supplementary material for: Assessing Guideline Knowledge Alignment of Large Language Models in Ophthalmology: A Preclinical Benchmarking Study on KLEx Evidence‐Based Guidelines
Source: J Ophthalmol. 2026 Aug 29;2026:4163980. doi: 10.1155/joph/4163980 (PMC13525438; doi:10.1155/joph/4163980)
Supplement: Supplementary file 1 [file JOPH-2026-4163980-s001.docx]

**1.1 Domain 1: Indications**

***1.1.1 Clinical Question 1* The influence of corneal biomechanical properties on the surgery (preoperative screening of keratoconus, selection of surgical options, surgical plans, and postoperative outcomes)**

1. Keratoconus is contra-indicated for corneal refractive surgery; preoperative screening of keratoconus and risk assessment are required; given the current technologies, assessment of corneal morphology combined with biomechanics is feasible to detect subclinical or forme fruste keratoconus; tomographic and biomechanical index (TBI), Corvis biomechanical index (CBI), and corneal resistance factor (CRF) are currently sensitive corneal biomechanical parameters (evidence quality: D; strong recommendation)
2. This surgery can preserve biomechanical properties postoperatively (evidence quality: B; conditional recommendation).
3. The thickness of the removed corneal tissue directly affects the corneal biomechanical strength, and lower thickness of removed corneal tissue is recommended; postoperative corneal biomechanical properties are associated with the optical zone diameter, and unreasonable expansion of the optical zone is not recommended (evidence quality: D; strong recommendation).
4. Corneal biomechanical properties influence the surgical predictability of refractive outcomes (evidence quality: D; conditional recommendation).

**1.2 Domain 2: Surgical Planning and Technique**

**1.2.1 Clinical Question 2 Safe ranges of corneal thicknesses in surgical planning (central corneal thickness [CCT], cap thickness, maximum lenticule thickness, and residual stromal thickness [RST])**

5. The calculation of the percentage of tissue alteration (PTA) for such procedures is mainly based on the removed corneal tissue (lenticular thickness) (evidence quality: C, conditional recommendation).
6. The exact cause leading to postoperative corneal ectasia remains unclear; however, when considering corneal thickness settings, an RST ≥280 μm and lenticule thickness index (LT index; ratio of maximum lenticule thickness to CCT) ≥28% could be used as reference values; caution should be taken when the RST is insufficient (<280 μm), commonly seen in thin corneas (<500 μm) and eyes with very high myopia (<-9 D); the procedure should be performed on the premise of much stricter preoperative screening and more diligent postoperative monitoring; given measurement error, an RST <250 μm should not be allowed (evidence quality: C; strong recommendation).
7. As appropriate, surgeons could reduce the cap thickness to ensure a sufficient RST in surgical planning; however, an extremely thin corneal cap (<100 μm) is not recommended (evidence quality: B; strong recommendation).

**1.2.2 Clinical Question 3 Setting ranges of the optical zone**

8. Large optical zones may result in less under-correction after surgery, especially in patients with high myopia, and small optical zones may increase the risk of refractive regression after surgery (evidence quality: D; conditional recommendation)
9. Larger optical zones are associated with fewer surgery-induced higher- order aberrations, and a large optical zone is recommended if the corneal thickness is sufficient(evidence quality: D; conditional recommendation).
10. Smaller optical zones have a smaller effect on postoperative corneal biomechanical strength (evidence quality: B; conditional recommendation).
11. For patients with thinner corneas or higher degrees of myopia, if the pupil size is small, an appropriate reduction in optical zone size (≥6.0 mm, except in exceptional cases) is recommended to prioritize adequate RST (Good Practice Statement; conditional recommendation).

**1.2.3 Clinical Question 4 Astigmatism correction ranges and auxiliary positioning Technique**

12.To date, the correction of astigmatism ranges from -0.25 D to -5.00 D; the higher the target astigmatism, the weaker the effects of correction postoperatively (evidence quality: B; conditional recommendation).

13.Correcting astigmatism with axis alignment and cyclotorsion compensation achieves better results postoperatively than without the use of the auxiliary positioning technique; when a large cyclotorsion angle is present, corresponding compensation is recommended to minimize alignment error and improve the accuracy of astigmatism correction (evidence quality: D; conditional recommendation)

**1.2.4 Clinical Question 5 The relationship between decentration and postoperative visual quality**

14.The centration locations used in clinical practice are mainly the coaxially sighted corneal light reflex (CSCLR) and entrance pupil center (EPC); both approaches have been found to achieve acceptable postoperative outcomes. However, CSCLR-centered procedures might be associated with better postoperative visual acuity, lower residual refractive error, and fewer induced higher-order aberrations, particularly coma (evidence quality: D; strong recommendation).

15. Some decentration within 0.2 mm will not cause a significant decrease in postoperative visual acuity and quality; in some cases, decentration within 0.5 mm has little impact on postoperative vision. As the amount of decentration further increases, visual acuity and quality are affected, even with decreased vision quality and increased residual astigmatism; therefore, accurate centration is crucial for surgery (evidence quality: C; strong recommendation).
16. The presence of a large angle kappa may lead to impaired postoperative visual acuity or visual quality; in this case, a corresponding adjusted centration is recommended during surgery to improve visual outcomes (evidence quality: B; strong recommendation).

**1.2.5 Clinical Question 6 Factors influencing the accuracy of preoperative refraction measurement**

17. Multiple factors can affect preoperative refraction, such as functional abnormalities (accommodation or binocular vision anomalies, visual fatigue, and inappropriate prescriptions for spectacles), organic diseases (nystagmus, strabismus, and corneal clarity), objective factors (optometrist experience), and subjective factors (repeatability of specific equipment), and all these factors are worth considering in preoperative refraction measurements (evidence quality: B; strong recommendation)
18. Accommodation is one of the most important factors affecting the precision of refraction measurement; the decision to perform a cycloplegic examination depends on a combination of factors, such as accommodative status and binocular visual function; when possible, cycloplegic refraction is recommended (evidence quality: B; strong recommendation).

**1.2.6 Clinical Question 7 Factors related to nomograms and adjusting strategies**

19. Nomogram adjustment is an essential part of the laser surgery procedure that directly affects surgical precision and predictability; the major factor related to nomogram settings is preoperative SE (or simple spherical values), while others include age, eye laterality, corneal curvature, diameter, and biomechanical properties, cap and lenticule thickness, CCT, RST, optical zone diameter, accommodation, and laser energy (evidence quality: B; strong recommendation).
20. The nomogram should be adjusted for multiple factors related to the patient, surgeon, and surgical environment; at present, adjusting strategies mainly include simple spherical and cylindrical modification, multivariate regression analysis, and artificial intelligence-based personalized adjustments (evidence quality: C; strong recommendation).

**1.3 Domain 3: Complications**

**1.3.1 Clinical Question 8 Prevention and management of intraoperative adverse events (difficult lenticule dissection, wrong plane dissection and difficult lenticule removal, incision tearing, and cap perforation)**

21. (preventive measures) Difficult lenticule dissection, improper laser energy, and severe OBL and black spots should be prevented and avoided. Additionally, inexperienced surgeons should avoid performing procedures on corneas with very low myopia or increase the minimum lenticule thickness as appropriate. To prevent wrong plane dissection, the upper and lower planes should first be differentiated at the incision site before lenticule dissection; surgeons should also be familiar with the signs of unintended posterior plane dissection and make prompt corrections when detecting abnormalities. To prevent cap perforation or tears, excessively thin caps should be avoided and surgeons should ensure good intraoperative fixation by requesting the patient’s cooperation to avoid sudden eye movements (evidence quality: C; strong recommendation)
22. (management principles) The lenticule should be carefully identified and dissected; if the lenticule cannot be found, a relatively sharp dissector should be used to search for the edge of the lenticule, and the magnification of the microscope should be increased or the built-in slit lamp should be used to identify the lenticule. OCT can also be applied when necessary; if the lenticule still cannot be found, the procedure should be postponed. In the event of cap perforations or tearing, the cornea must be tightly aligned, and a bandage contact lens is recommended (evidence quality: B; strong recommendation).

**1.3.2 Clinical Question 9 Prevention and management of suction loss**

23. (preventive measures) Preoperatively, patient education and fixation training should be reinforced; during the procedure, the patient’s head and eye positions should be appropriately adjusted, and patients should be instructed to remain relaxed and to maintain fixation; during suction, conjunctival tissue trapping and excess water on the ocular surface and conjunctival sac should be avoided, factors that may distract the patient’s attention should be eliminated, and the laser scanning mode should be modified by increasing spot and track separation to shorten the operative time (evidence quality: A; strong recommendation)
24. (management principles) If suction loss occurs during laser scanning at the periphery of the lower lenticule plane (< 10%), the original parameters should be retained, and redocking and reinitiating the procedure should be performed; if at the near-central (>10%) or central region of the lower plane, KLEx should be rescheduled or converted to other procedures. For suction loss during the side-cut, redocking and re-initiation should be performed with the option to increase the incision depth (e.g., 10 μm) or decrease the lenticule diameter (e.g., 0.2 mm). For suction loss during upper scanning (cap cutting) and incision creation, the original parameters should be retained, followed be redocking and continuing with the procedure; experienced surgeons could manually create the incision (evidence quality: B; strong recommendation)

**1.3.3 Clinical Question 10 Prevention and management of black spots**

25. (preventive principles) Prevention is primary, including strict adherence to patient selection, maintenance of a clean and properly humidified ocular surface and suction ring/patient interface, avoidance of repeated suction procedures, selection of appropriate laser energy (not too low) and scanning modes, and avoidance of abnormal laser output (evidence quality: B; strong recommendation).
26. (management principles) For small or peripheral black spots in the non- optical zone, the procedure should be continued with cautious dissection; for larger black spots, the procedure should be suspended and the causes should be determined. When necessary, reschedule the procedure or convert to other surgeries (evidence quality: B; strong recommendation)

**1.3.4 Clinical Question 11 Prevention and management of retained lenticule fragments**

27. (prevention measures) Standard surgical procedures are recommended;preoperatively, laser energy, surgery-related parameters, and temperature and humidity of the operating room should be set; after lenticule extraction, complete removal must be confirmed. Postoperatively, physicians should be vigilant for symptoms indicating retained lenticule fragments, such as poor visual acuity, severe irregular astigmatism, or abnormal corneal topography (evidence quality: B; strong recommendation).
28. (management principles) When retained lenticular tissue is discovered, it should be thoroughly removed, especially when in the optical zone; the minimal residual tissue in the peripheral areas that does not affect visual acuity can be followed up (evidence quality: B; strong recommendation).

**1.3.5 Clinical Question 12 Prevention and management of perioperative infections**

29. (preventive measures) Preoperatively, surgeons should strictly follow surgical indications, prophylactically administer antimicrobial drugs, and actively treat and control ocular surface diseases that may predispose patients to infection. Intraoperatively, they should strictly comply with aseptic practices and avoid excessive manipulation. Postoperatively, they should pay attention to ocular hygiene, rationally administer drugs with close follow-up, and attempt to promptly detect and treat any infections (evidence quality: B; strong recommendation).
30. (management principles) Physicians should identify the cause and the pathogen as early as possible and adopt anti-infection measures and practice rational administration of drugs to alleviate inflammatory reactions. If the infection is uncontrollable, the incision should be enlarged or opened as soon as possible, and the lesion should be removed in the intrastromal pocket followed by rinsing the pocket with antibiotics (evidence quality: A; strong recommendation)

**1.3.6 Clinical Question 13 Prevention and management of postoperative DLK**

31.(preventive measures) Preoperatively, the external eye should be carefully examined, the operative eye should be strictly cleaned and disinfected, disinfection methods for medical apparatus and instruments should be improved, and residual disinfectant should be avoided. During the procedure, physicians should select and set an appropriate laser energy, improve surgical skills with gentle operative manipulation, avoid repeated insertion and removal of instruments, change the incision position to prevent bleeding if necessary, and avoid oil and tear accumulation. Postoperatively, prophylactic glucocorticoids should be applied, with regular follow-up (evidence quality: B; strong recommendation)

32.(treatment principles) First, infection should be differentiated from inflammation, and routine local treatments should be applied; combined medications or interlamellar steroid rinsing should be used for patients with severe symptoms, and caution should be taken for interlamellar rinsing in late-onset and atypical cases (evidence quality: A; strong recommendation).

**1.3.7 Clinical Question 14 Causes, prevention, and treatment principles of delayed visual recovery and poor visual acuity after surgery**

33.(causes) Individual differences among patients, the occurrence of various intraoperative conditions, excessive manipulation, and poor laser stability can induce early postoperative corneal edema and healing reactions (evidence quality: A; strong recommendation).

34.(preventive measures) Preoperatively, surgical protocols must be carefully planned, especially for older patients and those with high myopia or unstable visual function. Intraoperatively, laser energy must be appropriately set, and excessively thin caps and excessive corneal manipulation should be avoided (evidence quality: A; strong recommendation)

35.(treatment principles)Visual acuity often improves gradually over time and with the subsiding of tissue edema. Corticosteroid or nonsteroidal anti-inflammatory eye drops should be used in cases with obvious corneal edema. Visual function training should be provided to patients with abnormal visual function; if their vision does not recover for a prolonged period of time with the identified cause of residual postoperative refractive error, enhancement surgery can be performed after ensuring a stable refraction status (evidence quality: A; strong recommendation).

**1.3.8 Clinical Question 15 Early diagnosis, prevention, and management of postoperative corneal ectasia**

36. (early diagnosis) For suspected postoperative corneal ectasia with visual decline and newly detected refractive errors, surgeons should promptly examine and monitor the corneal thickness and posterior corneal elevation with a close follow-up; early identification of postoperative ectasia is similar to that for subclinical or forme fruste keratoconus (described in Recommendation 1) (evidence quality: B; strong recommendation).
37. (preventive measures) Surgeons should avoid performing procedures on corneas with abnormal morphology with a close follow-up; postoperatively, patients should avoid eye rubbing, and physicians should pay attention to changes in posterior corneal elevation and evaluate long-term safety (evidence quality: B; strong recommendation)
38. (treatment principles)For corneal ectasia with a definite diagnosis with signs of progression and visual decline, timely interventions such as corneal cross-linking are recommended to halt disease progression; and rigid contact lens can be used to improve vision (evidence quality: B; strong recommendation)
