# Supplementary material for: Assessing Guideline Knowledge Alignment of Large Language Models in Ophthalmology: A Preclinical Benchmarking Study on KLEx Evidence‐Based Guidelines
Source: J Ophthalmol. 2026 Aug 29;2026:4163980. doi: 10.1155/joph/4163980 (PMC13525438; doi:10.1155/joph/4163980)
Supplement: Supplementary file 2 [file JOPH-2026-4163980-s002.docx]

For corneal ectasia with a definite diagnosis with signs of progression and visual decline, timely interventions such as corneal cross-linking are recommended to halt disease progression; and rigid contact lens can be used to improve vision.

This is a recommendation about ‘Early diagnosis, prevention, and management of postoperative corneal ectasia’ of Keratorefractive lenticule extraction(KLEx) surgery. Is this statement correct? Please provide a final score for this statement. The scores are quantified as “2”, “1”, “0”, “-1”, and “-2”. Different grades represent different trade-offs between the advantages and disadvantages and whether recommended in the present guidelines, as follows:

“2”indicates that the advantages significantly outweigh the disadvantages, strongly recommended;

“1” indicates that the advantages probably outweigh the disadvantages, weakly recommended;

“0”indicates equivalent or uncertain advantages over disadvantages, WITHOUT recommendation;

“-1” indicates that disadvantages probably outweigh advantages, weakly NOT recommended;

“-2” indicates that disadvantages significantly outweigh advantages, strongly NOT recommended.
